# Exfoliated MoS_2_ in Water without Additives

**DOI:** 10.1371/journal.pone.0154522

**Published:** 2016-04-27

**Authors:** Viviane Forsberg, Renyun Zhang, Joakim Bäckström, Christina Dahlström, Britta Andres, Magnus Norgren, Mattias Andersson, Magnus Hummelgård, Håkan Olin

**Affiliations:** Department of Natural Sciences, Mid Sweden University, Sundsvall, Sweden; RMIT University, AUSTRALIA

## Abstract

Many solution processing methods of exfoliation of layered materials have been studied during the last few years; most of them are based on organic solvents or rely on surfactants and other funtionalization agents. Pure water should be an ideal solvent, however, it is generally believed, based on solubility theories that stable dispersions of water could not be achieved and systematic studies are lacking. Here we describe the use of water as a solvent and the stabilization process involved therein. We introduce an exfoliation method of molybdenum disulfide (MoS_2_) in pure water at high concentration (i.e., 0.14 ± 0.01 g L^−1^). This was achieved by thinning the bulk MoS_2_ by mechanical exfoliation between sand papers and dispersing it by liquid exfoliation through probe sonication in water. We observed thin MoS_2_ nanosheets in water characterized by TEM, AFM and SEM images. The dimensions of the nanosheets were around 200 nm, the same range obtained in organic solvents. Electrophoretic mobility measurements indicated that electrical charges may be responsible for the stabilization of the dispersions. A probability decay equation was proposed to compare the stability of these dispersions with the ones reported in the literature. Water can be used as a solvent to disperse nanosheets and although the stability of the dispersions may not be as high as in organic solvents, the present method could be employed for a number of applications where the dispersions can be produced on site and organic solvents are not desirable.

## Introduction

Transition metal dichalcogenides (TMDs) in their bulk form have been known and studied for decades [1–5] but research around these materials has faced a revival during the past few years partly due to the advances in graphene research [6, 7]. The absence of a band gap in pristine graphene, which is also a layered material, further motivated studies of semiconductor materials such as molybdenum disulfide (MoS_2_)[8]. Metal atoms are sandwiched between two layers of chalcogen atoms in layered materials such as the TMD MoS_2_ where, for instance, strong covalent forces hold the individual atoms within each layer together and the layers are kept together by weaker van der Waals forces [7, 9]. During exfoliation these weaker forces are overcome, resulting in very thin films and possibly single nanosheets of the layered material. MoS_2_ is one of the most studied TMD and has mainly been employed in catalysis [4, 10] and as a lubricant [11]. Several novel properties appear when thinning down to a single or few nanosheets of the material [9]. The band gap shift from the near infrared to the visible range, for example, makes these materials especially interesting for optoelectronics [8] such as photovoltaic applications [12]. Bulk MoS_2_ has an indirect band gap of 1.2 eV [13] and by exfoliating the material the number of layers is reduced and the band gap can increase up to 1.9 eV [8] for single nanosheets, where for instance, a transition to a direct band gap semiconductor was observed [8].

Micromechanical cleavage [6] has been the main exfoliation method used for studies of single nanosheets of MoS_2_. A number of other exfoliation methods [14] and synthesis [14, 15] of thin films of MoS_2_ for low throughput application were also reported but just a few of them are scalable [9]. For applications where larger quantities of exfoliated nanosheets are needed, higher throughput methods are desirable [7]. Examples of such applications are the catalysts for hydrogen evolution reaction (HER) where MoS_2_ was reported to be an inexpensive alternative to platinum [16] and as mechanical reinforcement for polymers [17]. Besides, MoS_2_ nanosheets have been employed for the fabrication of highly flexible film transistors [18] with high mobility (12.5 cm^2^ V^−1^ s) and high on/off current ratio (10^5^), compared to a transistor based on a single nanosheet deposited by chemical vapor deposition which had mobility (200 cm^2^ V^−1^ s) and on/off current ratio (10^8^) [19].

The classical exfoliation method in water is lithium intercalation (LI) [20], and although the method gives a high population of single nanosheets [21], the process must be carried out under inert conditions due to the flammability of the lithium source, and the exfoliation process can only start after the intercalation process is ceased, which can take up to three days [21]. Besides, the intercalation process can cause a change in the crystal structure that consequently results in a shift from semiconductor to a metallic material [21] disabling semiconductor applications.

Therefore other liquid exfoliation methods have been employed by Coleman et al. [22]. A suitable solvent is needed as well as a mechanical force. For the mechanical part, sonication is the most common method to exfoliate layered materials, however, the method is not scalable and with limited efficiency. Therefore several methods have been developed to mechanically exfoliate layered materials including mortar and pestle [23], ball milling [24], sand paper [25], kitchen blenders [26], and shear mixers [27]. The sand paper method might be of interest due to the simple procedure and since it is possible to work on dry samples, the method should be very fast because of the resulting large shear forces, however, it is less well investigated compared to the other methods. For the solvent mainly two types are used, one using high boiling point organic solvents [22] and a second type using water with an added agent such as surfactants [28–30]. The agents used in the later method can be added directly to the dispersion during the liquid exfoliation step without changing the crystal structure [17, 22].

The problem with such approaches is that when those agents are removed, re-stacking of the nanosheets may occur [17]. The liquid exfoliation process in organic solvent was the process that achieved the highest concentration and most stable dispersions [17, 22, 24, 26, 29, 31]. However, removal of the solvent after application may be challenging due to their high boiling point and toxicity. Low boiling point organic solvents have been used, but the final throughput concentration was rather low [31, 32].

Another approach, to avoid high boiling point organic solvents, is to use low boiling point solvents such as ethanol [32] or isopropyl alcohol (IPA) [22, 33, 34]. Such ethanol mixture will have the same advantage as our water only approach and would not leave any residues. However, pure water is slightly simpler, less expensive, and more environmental friendly than an ethanol-water mixture. Pure water should thus be an ideal solvent due to simpler processing, no need for removing added agents, and low boiling point. However, it is generally believed, based on solubility theories [22, 35], that stable dispersions could not be achieved and no systematic study has been performed using only pure water.

Here, we present a water method of liquid exfoliation of MoS_2_. First, bulk MoS_2_ powder was pre-processed between sand papers, and no agents or intercalation components for dispersion stability were used. The second step was dispersion in water by sonication. In this way we were able to speed up the overall exfoliation process as compared to only sonication. We measured electrophoretic mobility to estimate the zeta potential (ZP), *ζ*. According to these values many particles were in the range of dispersion stability after liquid exfoliation. To quantify the stability we applied a first order reaction equation to data reported by previous authors [22, 24, 32] and compared to the present work.

Comparable to other solution processing methods, the nanosheets dimensions obtained by the present method were in the same order of magnitude and range. These dimensions were characterized by AFM and dynamic light scattering (DLS) measurements. Our conclusion was that water can be used as a solvent to disperse nanosheets. Although the stability was not as high as in organic solvents, this method could be used for a number of applications where the dispersions can be produced on site and organic solvents are not desirable.

## Materials and Methods

The MoS_2_ (Molybdenum IV sulfide) powder (99% < 2 μm) was purchased from Aldrich and used as supplied.

The orbital sander used to exfoliate the powder between the sand papers featured a nominal speed of 11000 rpm, (Meec Tools) and no external pressure was added. Sand papers with mesh size 2000 were employed following other authors’ [25] previous works. Sand papers with mesh size of 1000 or higher are also recommended for this method. We exfoliated the powder for 2 minutes, scraping the faces of the sand papers every 10 seconds with a spatula. The sand papers were attached, one to the sander and the other to a flat surface. The liquid exfoliation was performed using a Sonics Vibracell CV334, 750W probe unit equipped with a 13mm long step horn tip for volumes of up to 250 mL. The temperature of the dispersion during sonication was controlled by circulating 5°C water through a 100mL jacket glass vessel (Ace glass incorporated) connected to a heating circulating bath (Polyscience 801 heat circulator). The probe was set to operate 8 s and stand by for 2 s during the 60 min processing time to avoid excessive heating, solvent evaporation and damage to the converter.

We accessed force microscopy (AFM) imaging of the nanosheets deposited on silicon wafer using a Nanosurf Easyscan 2 operated in tapping mode for the statistical analysis of the nanosheets’ dimensions. For these AFM experiments we prepared MoS_2_ dispersions in water with initial concentration (C_i_ = 50 g L^−1^) and probe sonicated them for 50 min. The samples were centrifuged for 45 min at 1500rpm using a Hermle Z200A centrifuge to remove non-exfoliated particles.

Samples for AFM imaging of the nanosheets at higher resolution were collected from a C_i_ = 5 g L^−1^ dispersion that was centrifuged and decanted. This aliquota (which had a concentration of approximately C_f_ = 0.14 g L^−1^), was then centrifuged once again at 10000rpm (JouanA14) for 2 min to sediment all the particles. We then removed the water and re-dispersed the particles in 99,5% ethanol. We used a Dimension 3100 AFM (Digital Instruments) operated in tapping mode for these images.

The final concentration of the dispersions was measured by drying a known volume of the dispersion in a beaker of known mass at 100°C overnight and the remaining mass determined using a microbalance.

The transmission electron microscopy (TEM) images were accessed using a JEOL 2000FX operated at 160kV and the scanning electron microscopy (SEM) using a FEI Nova NanoSEM (450). SEM images of the sample surfaces were acquired in secondary electron imaging mode using 2 kV accelerating voltage and 5 mm working distance. Before image acquisition, the samples were gold sputtered for 20 s to obtain an electrically conductive surface.

The optical properties were evaluated by UV-vis absorption measurements using a UV-1800 Shimadzu spectrophotometer. The samples were left to settle in a bench for 30 days before measurement, when they were then decanted and diluted at a 1:9 ratio.

The stability of the dispersions was analysed by concentration and electrophoretic mobility measurements. We used a Malvern Zetasizer Nano instrument. The measurements were done shortly after sample preparation on samples that were and were not solution processed by liquid exfoliation. Another measurement of mobility was done for the same exfoliated sample 3.5 months later. The non exfoliated dispersions were at an initial concentration of C_i_ = 5 g L^−1^ and the exfoliated dispersion at C_f_ = 0.14 g L^−1^.

Dynamic light scattering (DLS) measurements were done to estimate the particle size distribution of the nanosheets before and after exfoliation using a Malvern Zetasizer Nano instrument for the same samples used to estimate the mobility.

Fourier Transform Infra Red (FTIR) measurements were done using a Nicolet 6700. The powder was collected by vacuum filtration of the liquid exfoliated dispersions onto a cellulose membrane. The liquid exfoliated powder was scraped from the surface of the membrane with an spatula. The mechanical exfoliated and bulk powder were measured without additional processing.

For the X-ray diffraction analysis we used a D2 Phaser (BRUKER) X-ray power diffraction station. The bulk powder and the exfoliated powder were deposited on a tape or silicon wafer, and the dispersions were deposited on nitrocellulose membrane filters through vacuum filtration.

## Results and Discussion

The exfoliation process consisted of two steps. The first one, mechanical exfoliation, we did using an orbital sander to exfoliate the bulk MoS_2_ powder for 2 min between sand papers. The second step was the liquid exfoliation, in which we used the processed powder from the first step to produce the dispersions, which were then liquid exfoliated by sonication.

**Fig 1:**
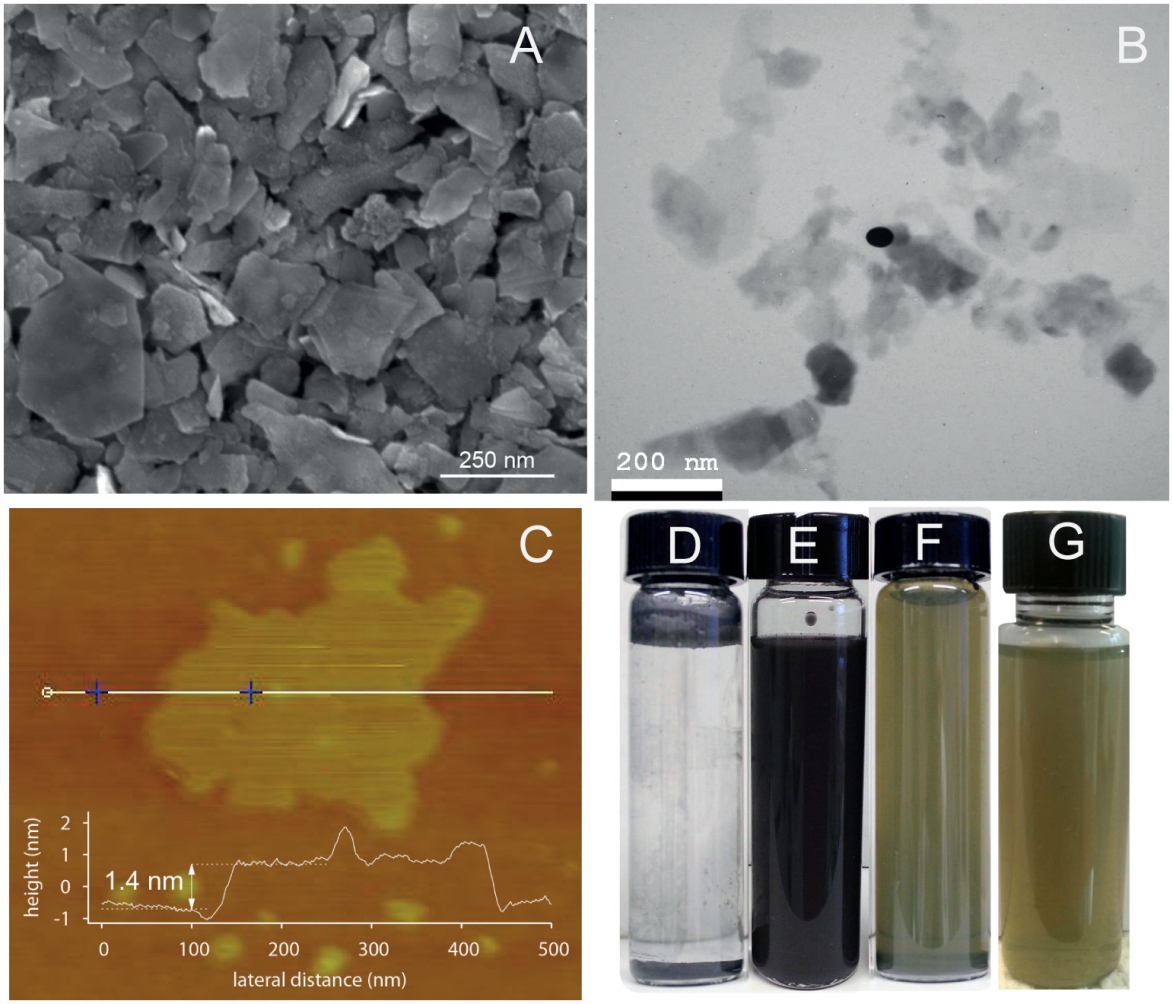
Nanosheets characterization for MoS_2_ dispersions in water. Fig 1-A, SEM image of vacuum filtrated dispersion onto polycarbonate membrane, Fig 1-B, TEM image of nanosheets, Fig 1-C, AFM image of a nanosheet deposited on silicon wafer with the thickness profile on the inset. Fig 1-D, is a photograph of the dispersion before exfoliation. Fig 1-E, Fig 1-F, and Fig 1-G are the photographs shortly after, 30 days and 4 months after exfoliation respectively.

We prepared a dispersion in water with initial concentration C_i_ = 5 g L^−1^. Liquid exfoliation was done by sonication for 60 min at 30% amplitude. Some samples were left to settle for 30 days in ambient temperature without centrifugation; a photograph of this dispersion is illustrated in Fig 1-F. Other samples were centrifuged for 45 min at 1500 rpm and the top 2/3 of the supernatant were decanted. The settings chosen for the sonication time, speed and centrifugation rate were set according to the optimal values reported in the literature [17, 22]. Fig 1-D is a photograph of the dispersion before liquid exfoliation and Fig 1-E to G shortly after exfoliation, 30 days and 120 days after exfoliation respectively.

**Fig 2:**
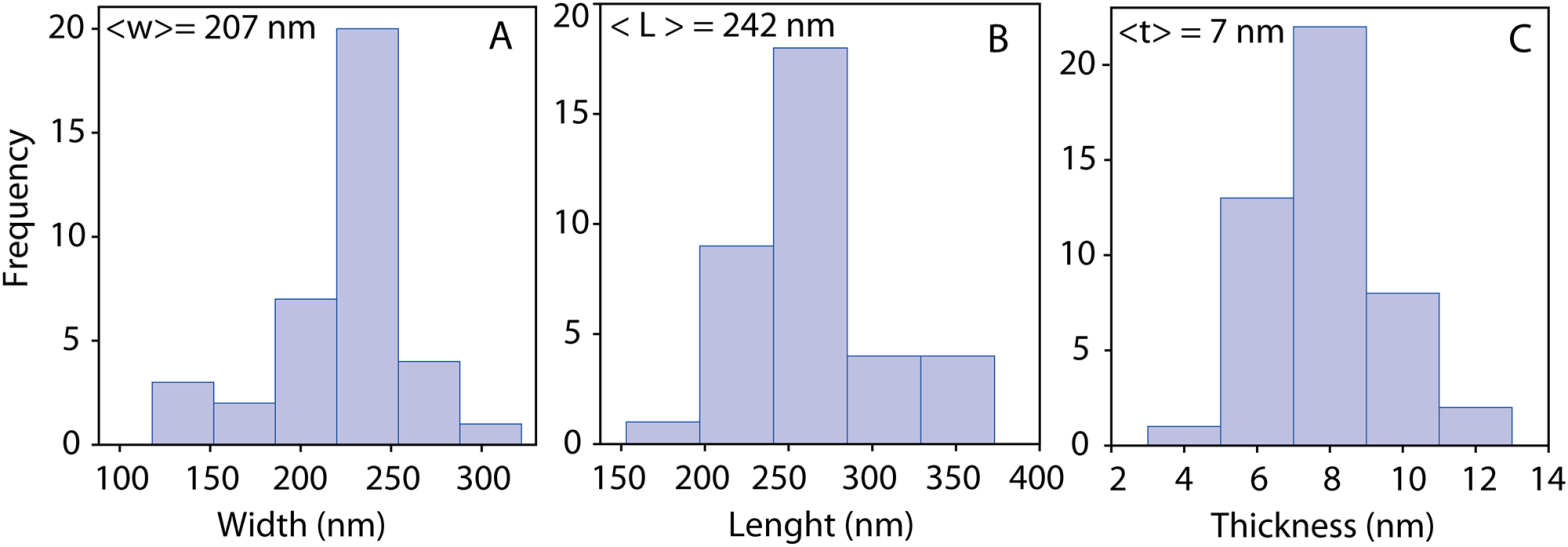
Statistical analysis for the average dimensions of nanosheets. Fig 2-A, width Fig 2-B, length and Fig 2-C, thickness for 50 nanosheets. The apparent average value is written on the left corner of each histogram.

**Fig 3:**
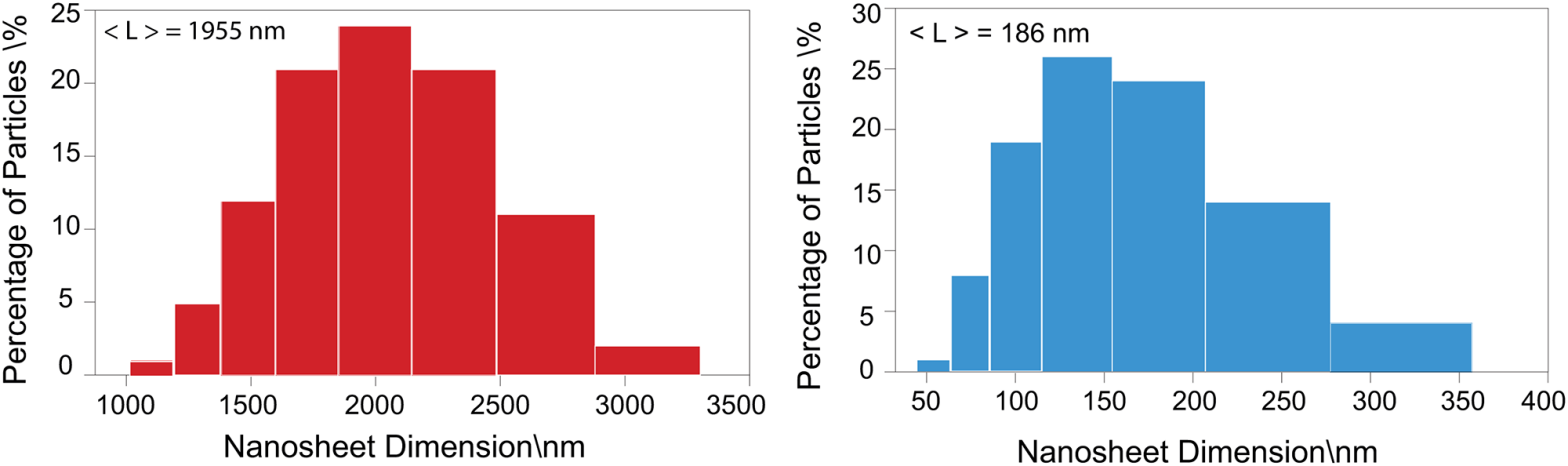
Particle size distribution (PSD) measurements for water dispersion before exfoliation on the left and, after liquid exfoliation on the right.

Microscopy (TEM, AFM and SEM) was performed to characterize the nanosheets dispersed in water. Thin, transparent and large nanosheets were observed by TEM imaging; an example was the one in Fig 1-B. This was also observed in the SEM images (see Fig 1-A). We attributed this transparency to the successful exfoliation of the bulk powder. The AFM image illustrated in Fig 1-C showed a very thin nanosheet in the inset. Statistical analysis of the measurements of the dimensions of the nanosheets, which were characterized by AFM images, are presented in the form of frequency histograms in Fig 2 indicating the observations of Fig 2-A width, Fig 2-B length and Fig 2-C thickness with the indicated average value among 50 nanosheets. The length was taken as the largest dimension of the nanosheet. The apparent value was 242 nm, which was comparable to the results obtained by the dynamic light scattering measurements (186 nm) presented in Fig 3 for a population of more than 1 million particles. The data bin sizes for the histograms illustrated in Fig 3 are in logarithmic scale due to the range of the particle sizes; in this way the bin sizes are proportional to the range of the particles. Our results from the AFM measurements indicated spherical particles; therefore we took these results as an approximation of the lateral dimensions, knowing that nanosheets typically do not have a spherical shape as we also observed on the high resolution AFM and TEM images presented here. We were aware that a statistical analysis using TEM would probably give better results [31] because the tip of the AFM can create some artifacts during imaging and the modeling behind the particle size distribution calculations considered spherical particles; however, as an estimation we consider these results valid based on the few measurements we performed on the nanosheets observed on the TEM images.

There are many solvents used for exfoliation [36], including dimethyl sulfoxide (DMSO) and dimethylformamide (DMF) but the best high boiling point solvent is NMP [17, 22] and we compare several NMP based exfoliation studies with our data in Table 1. In addition, surfactant dispersions [24, 26, 29] and low boiling point ethanol-water mixture is included for comparison [32].

**Table 1:** Liquid exfoliation (LE) methods for solution processing of MoS_2_ indicating the solvent used, if surfactant was employed, the final dispersion concentration, exfoliation time, and range or the average lateral nanosheet dimension reported according to AFM or TEM statistical measurements.

Method	Solvent	Surfactant [Yes/No]	Concentration [g L^−1^]	Exfoliation time [hour]	Size [nm]	Reference
LE	NMP	No	0.3	1	170	[22]
LE	NMP	No	7.6	50	700	[17]
LE	NMP	No	40	140	200	[17]
LE	EtOH/Water	No	0.018	8	100	[32]
LE	Water	Yes	0.25	0.5	280	[29]
SE	Water	Yes	0.4	10	85	[26]
ME+LE	Water	Yes	0.8	16	50−700	[24]
ME+LE	Water	No	0.14	1	242	[This work]

The acronyms refer to LE = liquid exfoliation, SE = shear exfoliation, ME = mechanical exfoliation, NMP = N-methyl pyrrolidone and EtOH = Ethanol.

The lateral size of the nanosheets produced by solution processing methods was still limited to around 300 nm as can be seen from the overview presented in Table 1 in agreement with our estimation of the lateral size. This size range is partly due to the size of clusters before exfoliation, which was already in the micrometer range as we estimated by DLS measurements presented in Fig 3, and also due to the sonication process, which tends to break the particles in a process called sonication scission [31]. O’Neill reported bigger particles according to TEM measurements [31]. The maximum reported concentration [31] achieved in organic solvents (40 g L^−1^) needed a long processing time of 140 h, and a reasonable concentration level can be achieved after 50 h but the half-time of the dispersion was not as good as for dispersions at lower initial concentrations [22].

In order to compare the data for stability, we applied a first-order reaction equation described by Eq 1. Where N_0_ is the initial dispersed concentration and N the concentration of exfoliated material in dispersion after a certain evaluation time t. *λ* is the decay rate and *t*_1/2_ is the time when N will be half of N_0_.

$$\begin{array}{c} N=N_{0}e^{-\lambda t} \end{array}$$ (1)

These data are presented in Table 2. The half-life value (t_1/2_) for a dispersion prepared with the present method and in the ethanol/water mixture was 23 h, a value that is rather low compared to the dispersions prepared in organic solvents but reasonable considering the advantages of having water as a solvent and no added agents to increase stability. Coleman and co-authors [22] describe a method to measure the stability over time using a home build apparatus, we refer these results in the first line of Table 2 were the initial concentration is taken as the concentration after centrifugation. The yield is normally very low for the dispersions of nanosheets and since we did not measured the stability over time we cannot directly compared to that. We then considered the initial concentration that was used to disperse the nanosheets in their work to compare with our results. Nevertheless, the half time is still higher in organic solvents than in water or water/ethanol mixtures.

**Table 2:** A probability decay equation (Eq 1) was applied to the stability data reported by other authors in the referenced papers and the stability data for the present work.

Method	Solvent	Surfactant [Yes/No]	N_0_/N	Evaluated time [hour]	t_1/2_ [hour]	Reference
LE	NMP	No	1, 1	600	6028	[22]*
LE	NMP	No	27	600	127	[22]
LE	EtOH/Water	Yes	167	168	23	[32]
ME+LE	Water	No	36	120	23	[This work]

*Initial concentration after centrifugation was considered in this presented data.

**Fig 4:**
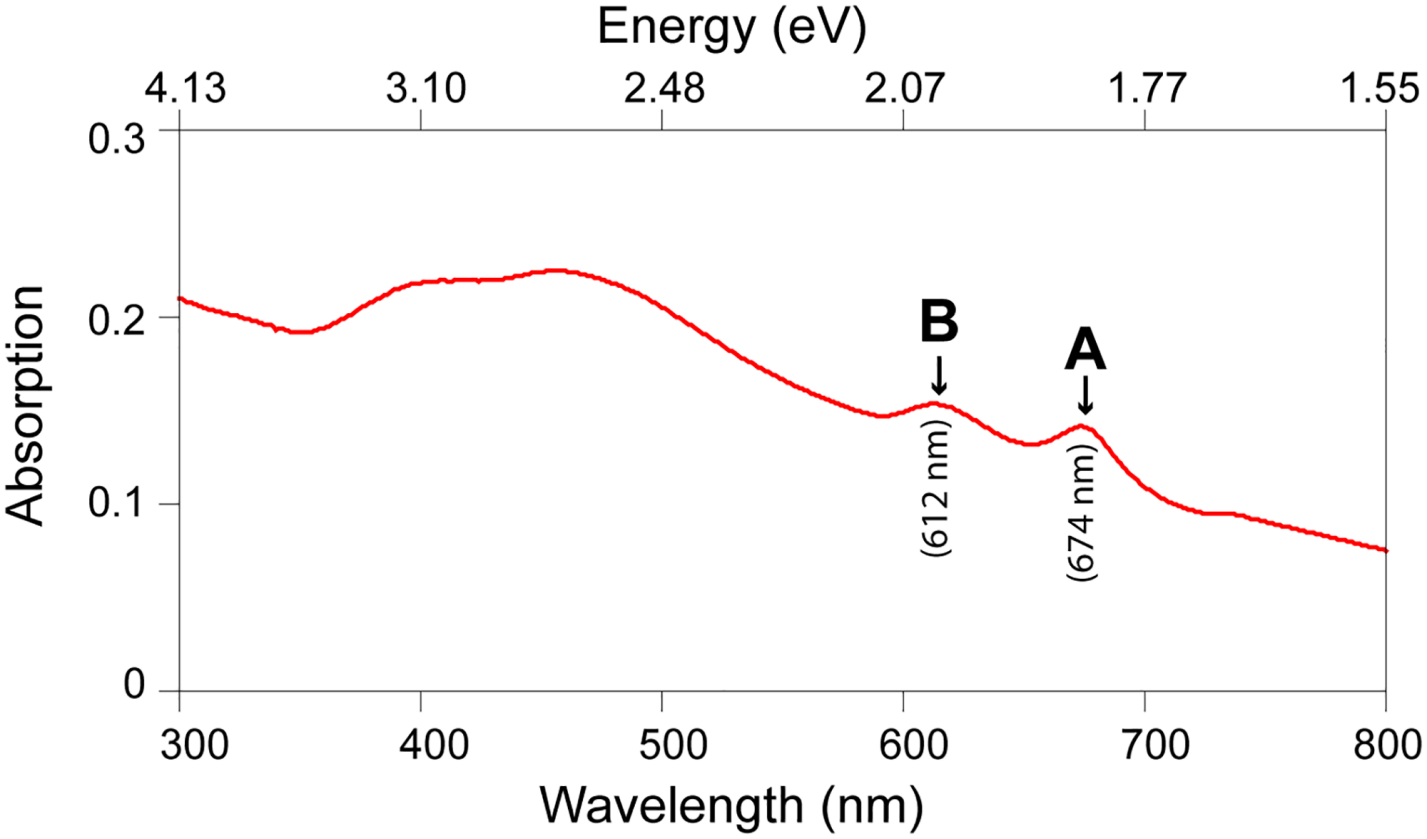
UV-Vis absorption spectrum for MoS_2_ dispersion in water.

The optical properties of typical MoS_2_ dispersions in water can be observed on the light absorption measurement presented in Fig 4. The A and B peaks at 674 and 621nm respectively, characteristic for MoS_2_ dispersions, were observed in the UV-Vis absorption measurements [10, 37].

**Fig 5:**
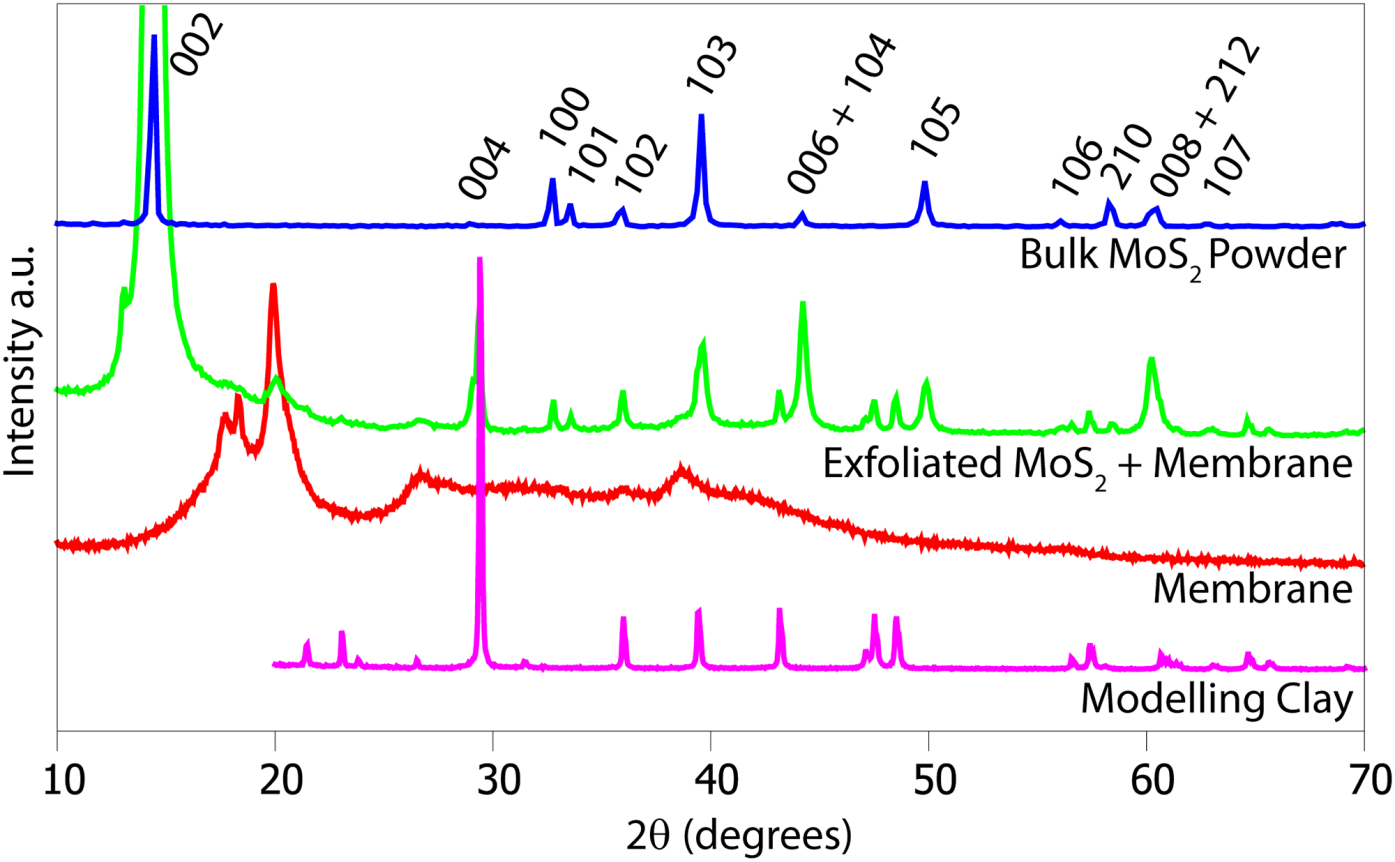
XRD measurements for the dispersion in water vacuum filtrated onto a cellulose membrane, the bulk powder deposited on a silicon wafer, the cellulose membrane filter and the modelling clay used to fix the sample onto the measurement container. The data for measurements were offset on the vertical axis for clarity. The Miller indexes for each reflexion are indicated for the bulk powder measurement [39].

**Fig 6:**
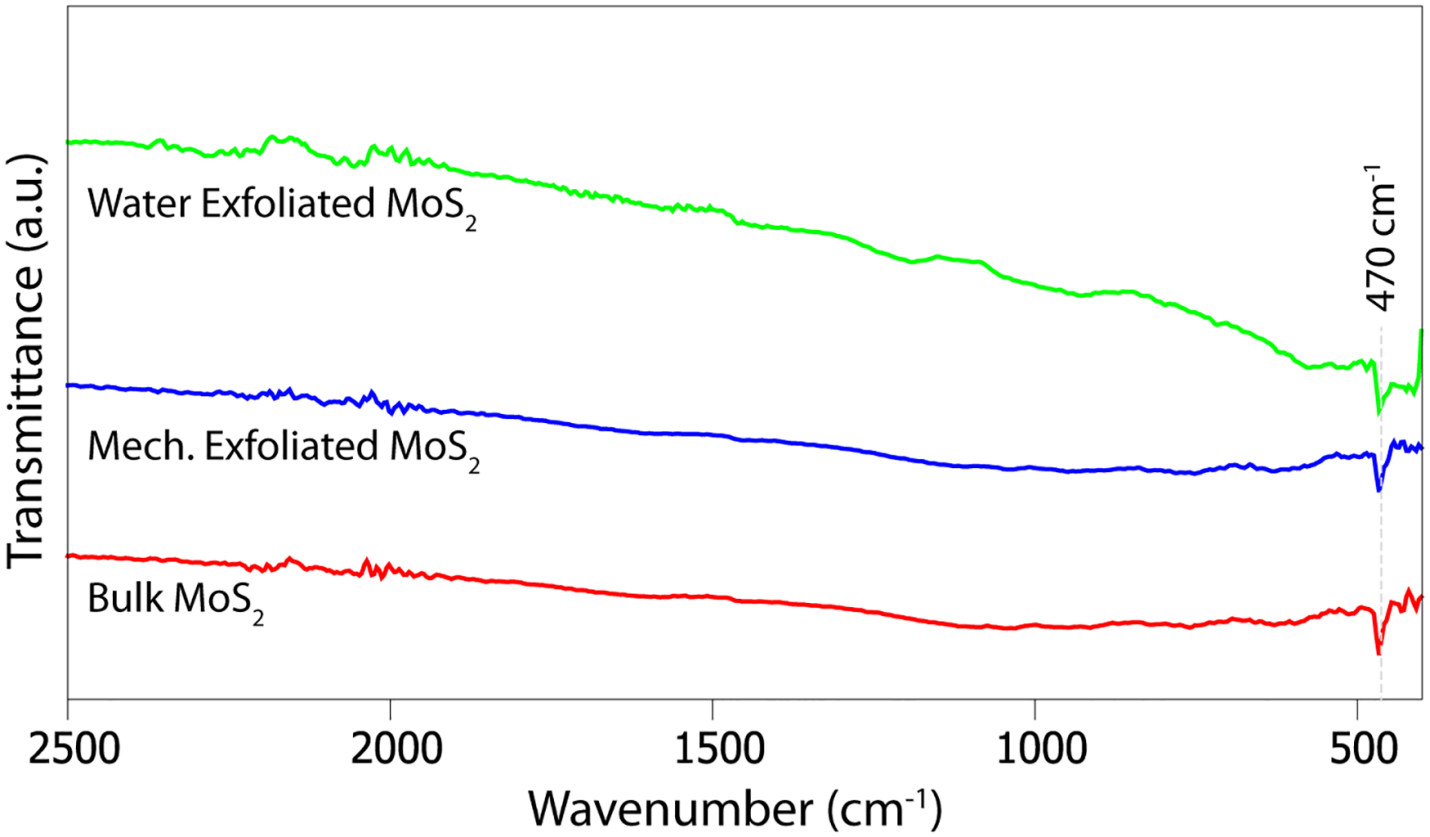
FTIR spectrum for MoS_2_. The data for measurements were offset on the vertical axis for clarity.

To evaluate the crystal structure of the nanosheets before and after the exfoliation steps, we performed X-ray diffraction (XRD) analysis on the powders and the dispersions deposited onto filter membranes by vacuum filtration (see Fig 5). We observed the standard peaks for molybdenite (MoS_2_)[38] and did not observe any peaks of molybdenum oxides such as MoO_3_[39] and MoO_2_[39]. The samples were attached onto the glass measurement tray with modeling clay; therefore we also presented the measurement for the clay itself. FTIR spectra measurements were performed to complement these XRD measurements for the liquid exfoliated, the mechanical exfoliated and the bulk MoS_2_ powders. The liquid exfoliated powder sample was obtained by vacuum filtration of the dispersion onto a cellulose membrane. The peak at 470 cm^−1^ in the far infrared (FIR) region observed in Fig 6, which is characteristic of MoS_2_ [40] was observed for all three measurements and no peaks for the oxides were observed.

We attributed the stability of the dispersions to the charges created during the liquid exfoliation process. We identified these charges by electrophoretic mobility measurements. Electrostatic stabilization can be quantified by measurements of zeta potential, which for instance, were estimated by the electrophoretic mobility measurements. The electrophoretic mobility measurements employed a laser interferometric technique (Zeta Sizer Nano Series Operating Instructions), which enabled the calculation of electrophoretic mobility to estimate the zeta potential; such a technique is called M3-PALS (Phase Analysis Light Scattering). Once we measured the mobility, the apparent zeta potential could be estimated using the Smoluchowski equation [41]. Where *ζ* is the apparent zeta potential, *η* is the viscosity of the dispersion liquid, *μ* is the electrophoretic mobility and *ε* is the solution permittivity.

$$\begin{array}{c} \zeta =\eta \mu /\varepsilon \end{array}$$ (2)

This method was derived for spherical particles; however, it can be used as an estimation for non-spherical particles as did other authors [42] to within 20% of the true value [30].

**Fig 7:**
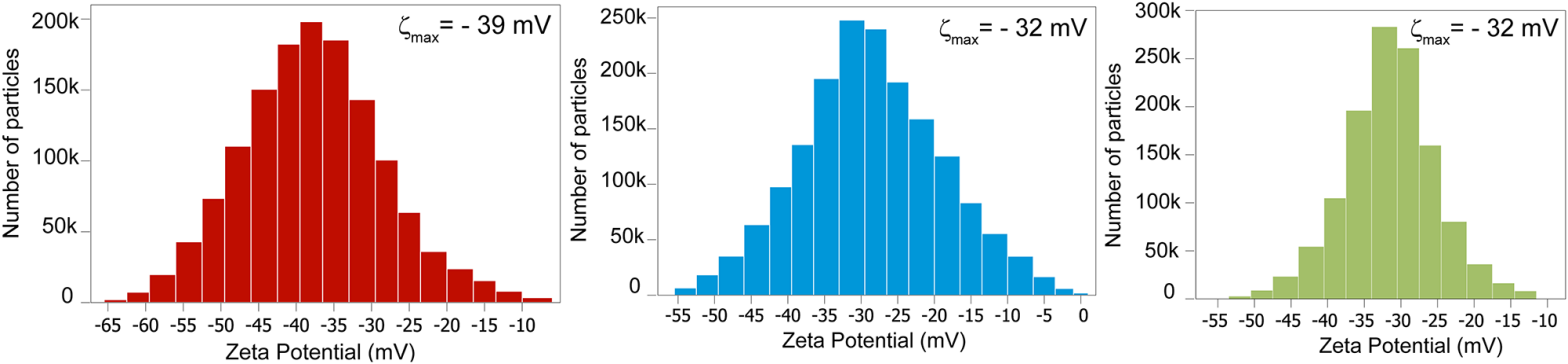
Electrophoretic mobility measurements and estimated zeta potential for water dispersion before exfoliation on the left, dispersion measured right after liquid exfoliation on the middle and, on the right dispersion measured after 30 days of liquid exfoliation. The number of particles exceeded 1 million on each measurement. The same measurement cell and sample were used for the PSD measurements.

If all of the particles have a large negative or large positive charge, they will repel each other and there is dispersion stability. If not, when |*ζ*| = 25 mV [43] the forces repelling these particles are very small, almost negligible, so they will come in contact and the dispersion will be unstable. According to the ZP distribution in Fig 7, most of the particles were in the range of dispersion stability after liquid exfoliation and a few percentage were not, which means that some sedimentation may still occur. During exfoliation a higher specific surface area on the particles was created, which means that to stabilize the dispersion more charges are necessary to cover these new surfaces otherwise these particles may sediment. The apparent zeta potential for the exfoliated dispersion (-32 ± 2 mV) and for the non exfoliated one (-39 ± 3 mV) indicated that charges were present in the systems that were accounting for the stability of the dispersions.

## Conclusions

In this study we introduced a method to disperse MoS_2_ in water involving mechanical and liquid exfoliation. The nanosheets were successfully exfoliated according to the AFM and TEM imaging. The dimensions that we obtained were in the same range of the liquid processed MoS_2_ dispersions reported by other authors [22, 31], and the concentration achieved by this method was half of the reported concentration achieved by liquid exfoliation in organic solvents at the same liquid exfoliation conditions [22]. Although the half time t_1/2_ of the dispersions in water were much lower than the ones prepared according to other methods [22] (see Table 1), we accounted for the advantages of using an environmental friendly solvent and no additional chemicals for the dispersion stability. The present method can be employed for large scale production of nanosheets at lower costs and in more environmental friendly conditions as if organic solvents are to be employed in conditions that may overcome the drawback of the lower stability of the dispersions in water only without additives. Nevertheless, we observed that even 4 months after the sample preparation, there were still particles dispersed on a sample prepared by the presented method.
